# NCAPD3 enhances Warburg effect through c-myc and E2F1 and promotes the occurrence and progression of colorectal cancer

**DOI:** 10.1186/s13046-022-02412-3

**Published:** 2022-06-11

**Authors:** Zuolei Jing, Qianmei Liu, Xinyuan He, Zhirong Jia, Zhizhong Xu, Bolin Yang, Ping Liu

**Affiliations:** 1 College of Life Sciences, Nanjing Normal University, No. 1 Wenyuan Road, Nanjing, 210023 Jiangsu PR China; 2 Department of Colorectal Surgery, Jiangsu Province Hospital of Chinese Medicine, Affiliated Hospital of Nanjing University of Chinese Medicine, No. 155 Hanzhong Road, Nanjing, 210029 Jiangsu PR China

**Keywords:** NCAPD3, Warburg effect, Colorectal cancer, E2F1, c-Myc

## Abstract

**Background:**

NCAPD3 is one of the three non-SMC subunits of condensin II complex, which plays an important role in the chromosome condensation and segregation during mitosis. Notably, elevated levels of NCAPD3 are found in many somatic cancers. However, the clinical role, biological functions of NCAPD3 in cancers especially in colorectal cancer (CRC) and the underlying molecular mechanisms remain poorly elucidated.

**Methods:**

Clinical CRC and adjacent normal tissues were used to confirm the expression of NCAPD3. The association of NCAPD3 expression with clinicopathological characteristics and patient outcomes were analyzed by using online database. In vivo subcutaneous tumor xenograft model, *NCAPD3* gene knockout following azoxymethane (AOM)/dextran sodium sulfate (DSS)-induced tumor mouse model, Co-IP, western blot, qRT-PCR, IHC, ChIP assays and cell functional assays were used to investigate the biological functions of NCAPD3 in CRC and the underlying molecular mechanisms.

**Results:**

NCAPD3 was overexpressed in CRC tissues and positively correlated with poor prognosis of CRC patients. NCAPD3 knockout suppressed CRC development in AOM/DSS induced and xenograft mice models. Moreover, we found that NCAPD3 promoted aerobic glycolysis in CRC. Mechanistically, NCAPD3 up-regulated the level of c-Myc and interacted with c-Myc to recruit more c-Myc to the gene promoter of its downstream glycolytic regulators GLUT1, HK2, ENO1, PKM2 and LDHA, and finally enhanced cellular aerobic glycolysis. Also, NCAPD3 increased the level of E2F1 and interacted with E2F1 to recruit more E2F1 to the promoter regions of PDK1 and PDK3 genes, which resulted in the inhibition of PDH activity and TCA cycle.

**Conclusions:**

Our data demonstrated that NCAPD3 promoted glucose metabolism reprogramming and enhanced Warburg effect in colorectal tumorigenesis and CRC progression. These findings reveal a novel mechanism underlying NCAPD3 mediated CRC cell growth and provide new targets for CRC treatment.

**Supplementary Information:**

The online version contains supplementary material available at 10.1186/s13046-022-02412-3.

## Background

Colorectal cancer (CRC) accounts for approximately 1 in 10 cancer cases and cancer-related deaths worldwide. It ranks third in terms of incidence and second in terms of mortality [1]. The incidence of CRC worldwide is predicted to increase to 2.5 million new cases in 2035 [2]. Although tremendous achievements have been made in surgery, immunotherapy, stereotactic radiotherapy and new chemotherapy drugs, the 5-year relative survival rate of CRC patients has not changed significantly in the past decades [3]. Hence, more researches on the pathological mechanisms of CRC are urgently needed to discover and develop effective biomarkers and new therapeutic targets for the diagnosis and treatment of CRC in clinic.

Non-SMC Condensin II Complex Subunit D3 (NCAPD3) is a subunit of the Condensin II complex, which is basically responsible for chromosome condensation and segregation during meiosis and mitosis [4]. In *Drosophila* and human intestinal epithelium, NCAPD3 plays a key role in the antibacterial effect of innate immunity by promoting the formation of interferon activated translation inhibitor (GAIT) complex and upregulating the genes required for innate immunity after systemic bacterial infection, or down-regulating the transcription of genes encoding amino acid transporters (SLC7A5 and SLC3A2) [5, 6]. Mutations in condensin subunits (NCAPD2, NCAPH, or NCAPD3) result in microcephaly due to impaired DNA decatenation [7]. Notably, elevated levels and frequently mutations of NCAPD3 are found in many somatic cancers [8]. There is a significant negative correlation between the expression level of NCAPD3 and overall survival in pancreatic ductal adenocarcinoma [9]. In prostate cancer, NCAPD3 is identified as a new biomarker for subtype-1 tumors that improves prognostication [10]. Our previous study finds that NCAPD3 is an androgen/androgen receptor (AR) axis-targeted gene and is involved in AR-promoted progression of prostate cancer [11].

Reprogramming of energy metabolism has long been recognized as one of the hallmarks of cancer [12]. Even in the presence of abundant oxygen, many cancer cells metabolize glucose by aerobic glycolysis instead of oxidative phosphorylation, a phenomenon known as the ‘‘Warburg effect’’ [13, 14]. It has been confirmed that this shift in cellular glucose metabolism promotes tumorigenesis through rapid ATP production, increased biosynthesis of macromolecules and maintenance of redox homeostasis [15]. Current research indicates that the driving mechanism of metabolic reprogramming mainly includes proto-oncogene activation or tumor suppressor inactivation, abnormal expression of metabolic enzymes, and continuous activation of signaling pathways such as PI3K/PKB, mTOR, and AMPK [16].

c-Myc and E2F1 are two important transcription factors and play the key roles in many cellular physiological processes including cell growth, cell cycle, proliferation and metastasis in multiple cancers [17, 18]. c-Myc is reported as an oncogene and promotes tumor proliferation by increasing the glycolytic activity of cancer cells under normoxic conditions [19]. It has been well-documented that c-Myc promotes glycolysis through transcriptionally upregulating glucose metabolism genes such as glucose transporter 1 (GLUT1), hexokinase 2 (HK2), pyruvate kinase M2 (PKM2), enolase 1 (ENO1) and lactate dehydrogenase A (LDHA) [20, 21]. E2F1 is a member of E2F transcriptional factor family and directly influences a number of biological processes, including cell cycle, cell proliferation, apoptosis, migration and DNA-damage response by regulating hundreds of its target genes [22, 23]. Recent studies implicate that E2F1 regulates mitochondrial function and loss of such regulation results in severe mitochondrial defects [24]. KDM4A-E2F1 complex regulates metabolism by upregulating pyruvate dehydrogenase kinase 1 (PDK1) and pyruvate dehydrogenase kinase 3 (PDK3), thereby promoting the switch of oxidative phosphorylation to glycolytic metabolism in prostate cancer cells [25].

In this study, it’s found for the first time that NCAPD3 was higher expression in CRC and functioned as an oncogene to promote the occurrence and progression of CRC by switching glucose metabolism from aerobic respiration to glycolysis (Warburg effect). From our data, NCAPD3 knockout (NCAPD3^±^) suppressed the colorectal tumorigenesis of mice induced by the azoxymethane/dextran sodium sulfate (AOM/DSS). Overexpression of NCAPD3 not only upregulated the levels of c-Myc and E2F1, but also presented a dose-dependent interaction between NCAPD3 and c-Myc or E2F1, and finally led to the increased transcription of c-Myc or E2F1 target genes. By which, NCAPD3 promoted aerobic glycolysis and inhibited TCA cycle, resulting in the glucose metabolism reprogramming and the enhancement of glycolytic fluxes and Warburg effect in CRC cells. Altogether, our study highlighted a novel role of NCAPD3 in glycose metabolism and development of CRC, suggesting a possible target for CRC diagnosis and treatment in clinic.

## Methods

### Clinical specimen

Clinical specimens including tumor tissues and matched adjacent nontumor tissues were obtained from the patients with CRC who underwent surgery in the Affiliated Hospital of Nanjing University of Chinese Medicine (Nanjing, China). All patients provided the informed consent for collected samples and subsequent analysis. Normal adjacent non-tumor tissues were collected by a distance of at least 5 cm from the cancer lesions. The samples were snap-frozen in liquid nitrogen or formalin-fixed and paraffin embedded for subsequent experiments. Our study got approval of the Committees for the Ethical Review at the Affiliated Hospital of Nanjing University of Chinese Medicine.

### Cell culture, transfection and generation of NCAPD3 knockdown or overexpressed stable clones

Human CRC cell lines (RKO, HCT116, SW480) and normal colonic epithelial cell line FHC were purchased from National Collection of Authenticated Cell Cultures (Shanghai, China) and cultured according to the manufacturer's instructions. Transient cell transfections of siRNA or plasmid were performed with Lipofectamine 2000 Transfection Reagent (Invitrogen, Carlsbad, CA, USA) according to the manufacturer's instructions. The siRNA oligonucleotides targeting NCAPD3, c-Myc, E2F1 and control siRNA were purchased from GenePharma (Shanghai, China) and listed in Supplementary Table S1.

The detection in three colorectal cancer cell lines showed that NCAPD3 had the lowest level in HCT116 cells and the highest level in SW480 cells. Therefore, NCAPD3 stable knockdown in SW480 cells with shNCAPD3 plasmid (purchased from Shanghai GeneChem Co., Ltd., Shanghai, China) and NCAPD3 stable overexpression in HCT116 cells with lentiviruses infection were carried out. Briefly, shNCAPD3 plasmid or lentiviruses contained lentiviral vectors (CMV-MCS-3FLAG-EF1-ZsGreen1-T2A-puromycin, purchased from Shanghai GeneChem Co., Ltd., Shanghai, China) harboring NCAPD3 were transfected/infected into cells with Lipofectamine 2000 or HitransGP (Shanghai Genechem Co., Ltd., Shanghai, China), respectively. The transfected/infected cells were selected in medium containing 2 µg/mL puromycin. Stable clones grew after about 2 weeks of selection and were picked up for amplification, as well as identified using RT-PCR and western-blot assays.

### Antibodies and reagents

All detailed information is shown in Supplementary Table S2.

### CCK-8, colony formation and wound-healing assays

Experimental details were performed as described previously [26]. The relative migration distance was calculated by the following formula: percentage of wound closure (%) = 100(A-B)/A, A and B representing the width of cell scratches before and after incubation, respectively.

### Real-time PCR assay

Total RNA was extracted using TRIzol reagent (TaKaRa, Shiga, Japan) and cDNA was synthesized using PrimeScript RT-PCR Kit (TaKaRa, Shiga, Japan). Real-time quantitative PCR was performed using SYBR Green Master Mix (TaKaRa, Shiga, Japan) on a LightCycler 96 Detection System (Roche, Mannheim, Germany). The expression level of target mRNA was analyzed with the 2^−∆∆Ct^ method, following normalization with beta-actin gene. Primer sequences were listed in Supplementary Table S3.

### RNA-seq analysis

Total RNA was isolated from 3 paired CRC tissues (tumor tissues and matched nontumorous tissues) using Trizol reagent (TaKaRa, Shiga, Japan) according to the manufacturer's protocol. Further library preparation for strand-specific RNA-seq was carried out by poly(A) selection with the KAPA mRNA Capture Beads (KAPA), and fragmented to about 300–400 nucleotides (nt) in length, then all subsequent steps were performed according to a KAPA Stranded mRNA-Seq Kit Illumina platform (KK8421) and sequenced on an Illumina HiSeq Xten system to generate 150 bp pair-end reads at LC Biotech in Hangzhou, China. Gene expression level and transcript abundances were calculated using the FPKM (fragments per kilobase of transcript per million mapped reads) method.

### Pyruvate assay, lactate assay and ATP assay

Cellular pyruvate, lactate and ATP were measured with a pyruvate colorimetric assay kit, a lactate colorimetric assay kit and an ATP assay kit (Solarbio, Beijing, China), correspondingly. Briefly, cells (5 × 10^6^) were collected and washed with ice-cold PBS, and then lysed and extracted with the buffers of corresponding kit according to the instructions. The metabolites pyruvate, lactate and ATP were determined according to the manufacturer's instructions.

### LDH (lactate dehydrogenase) and PDH (pyruvate dehydrogenase) enzyme activity assays

LDH and PDH activity were measured using the LDH and PDH Enzyme Activity Assay Kits (Solarbio, Beijing, China) following manufacturer's protocols. Cells (5 × 10^6^) were collected and extracted with buffer of the corresponding kit. The optical density was measured spectrophotometrically using a microplate reader (Bio-Rad, Hercules, CA, USA).

### Glucose uptake assay

Cells treated as indicated in figures were incubated with a fluorescent deoxy-glucose analog 2-NBDG (2-(N-(7-Nitrobenz-2-oxa-1,3-diazol-4-yl)-Amino)-2-Deoxyglucose, APExBIO, Houston, TX, USA) in glucose-free medium for 1 h at 37 °C, followed by washing twice with PBS. Then, cells were collected and lysed with the lysis buffer containing 0.1% Triton X 100. The fluorescence was measured using microplate reader at excitation 485 nm and emission 535 nm.

### Assays of immunoprecipitation and western blotting

Experimental details were followed as described previously [26]. Signal was detected using an enhanced chemiluminescence detection kit (Tanon, shanghai, China) and quantified using Gel Imaging System with GIS ID Analysis Software v4.1.5 (Tanon, Shanghai, China).

### Chromatin immunoprecipitation-PCR assay

Chromatin immunoprecipitation (ChIP) assay was performed using the ChIP assay kit (Beyotime, Beijing, China) according to the manufacturer's instructions. In brief, cells were cross-linked with 1% formaldehyde for 10 min at 37 °C and the chromatin was sonicated to generate DNA fragments of 200–500 bp. Antibodies of c-Myc and E2F1 (IgG as the control) were utilized to precipitate the cross-linked protein-DNA complexes. The chromatin DNA was extracted using DNA Purification Kit (Beyotime, Beijing, China) and the samples were subjected to quantitative PCR analysis with specific primers. The primer sequences were listed in Supplementary Table S4.

### *In vivo* experimental assay of transplanted tumor in nude mice

NCAPD3-stable overexpression HCT116 cells (2 × 10^6^) or NCAPD3-stable knockdown SW480 (3 × 10^6^) cells and their respective control cells were resuspended in 100 μL PBS and injected subcutaneously into the left axillae of 5-week-old male BALB/c nude mice (purchased from GemPharmatech Co., Ltd., Nanjing, China) and then maintained under pathogen-free conditions. Tumor-bearing mice were treated with 2-Deoxy-D-glucose (2-DG, a glycolytic inhibitor, acts as a competitive inhibitor of the glucose metabolism) at a dose of 600 mg/kg body weight or saline as a control by intraperitoneal injection every other day. Four weeks after injection, mice were sacrificed and the xenograft tumors were isolated, weighed and applied to subsequent detection.

For lung metastasis mouse model, NCAPD3-stable overexpression HCT116 cells or control cells (2 × 10^6^ in 0.1 mL PBS) were injected via tail vein into 5-week-old male BALB/c nude mice. All mice were sacrificed 7 weeks after injection, and lung tissues were harvested and fixed in 4% paraformaldehyde.

### NCAPD3 knockout mice and AOM/DSS-induced *in vivo* CRC model

NCAPD3^±^ mice with C57BL/6 N background were generated by Cyagen Biosciences (Guangzhou, China) using CRISPR/Cas9 method. Genotyping of knockout mice were carried out with PCR (polymerase chain reaction) and all mice were housed under specific pathogen-free conditions. Intestinal tumors were induced by azoxymethane and dextran sulfate sodium according to our previous report [26]. All animal studies were approved by the Experimental Animal Ethics Committee of Nanjing Normal University and handled following the National Guidelines for Animal Usage in Research (China).

### Immunohistochemistry (IHC) assay

IHC studies were performed on formalin fixed de-paraffinized sections as previously described [26]. Images were photographed with microscope (Nikon, Tokyo, Japan).

### Statistical analysis

Statistical analysis was performed using SPSS version 17.0. A two-tailed, unpaired, or paired Student’s *t*-test was used to compare the variables of two groups, and one-way or two-way ANOVA were performed for multi-group comparisons. Data were presented as the mean ± standard deviation. All data were obtained from at least three independent experiments. *P*-values < 0.05 was considered statistically significant. *P < 0.05, **P < 0.01, ***P < 0.001.

## Results

### NCAPD3 was overexpressed in CRC cells and clinical specimens

**Fig. 1:**
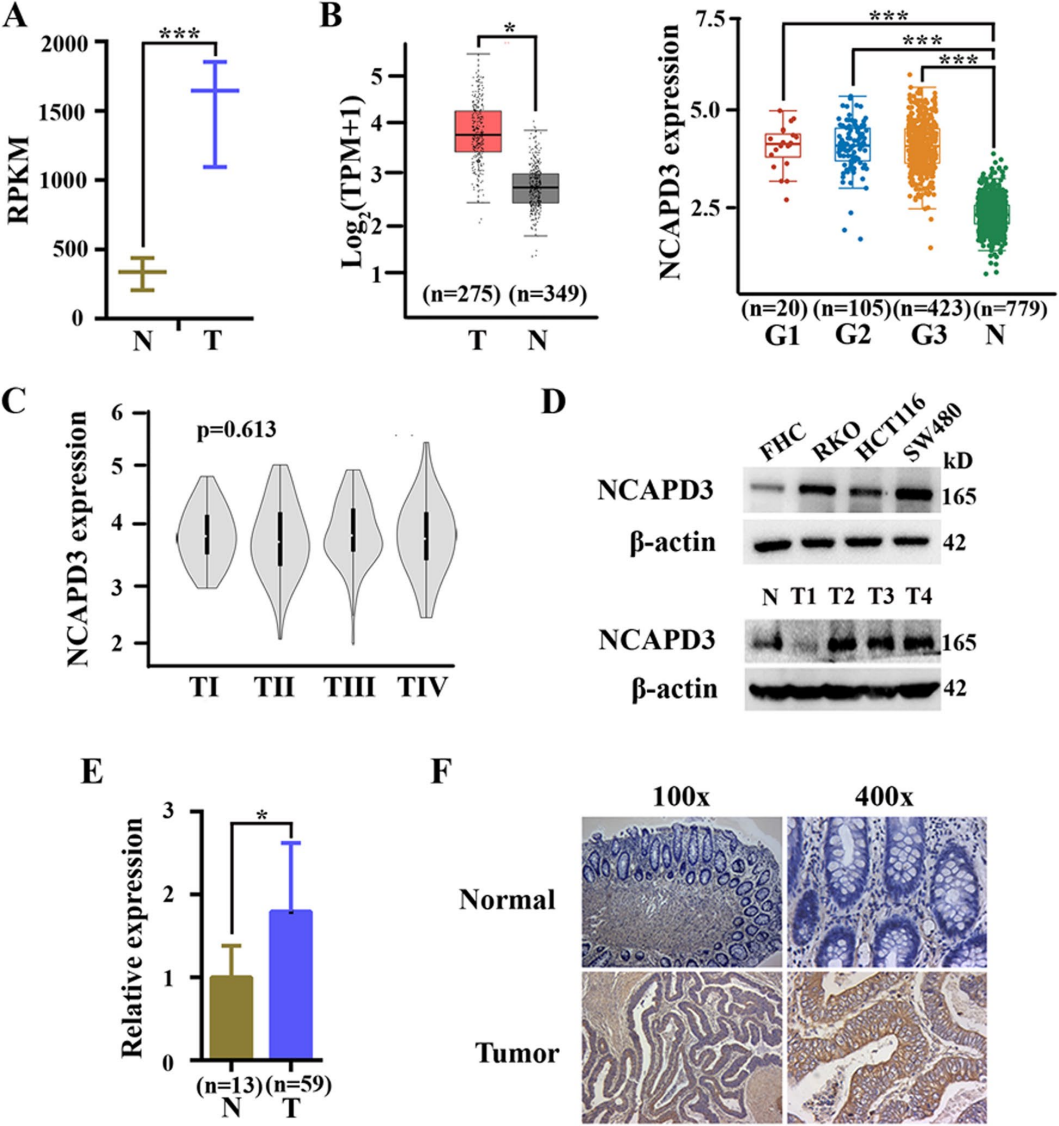
NCAPD3 was higher expression in human CRC. **A** Expressions of NCAPD3 in CRC tissues and corresponding non-tumor normal tissues of clinic specimen by analyzing our RNA-seq data. **B** The levels of NCAPD3 mRNA in CRC tissues and normal tissues were analyzed by using GEPIA (left) and Assistant for Clinical Bioinformatics (right) web tools. G1, G2, G3 represent different tumor grades. **C** The level of NCAPD3 at different tumor stages were conducted by using online database. **D** Protein levels of NCAPD3 in human normal colorectal mucosal cell line FHC, CRC cell lines (RKO, HCT116 and SW480) and clinic specimen (including CRC tissues and normal colorectal tissues) were detected by Western blot assay. **E** qRT-PCR analysis of mRNA expression in 59 human CRC tissues and 13 normal colorectal tissues. **F** Immunohistochemistry (IHC) detection of NCAPD3 was carried out in CRC tissues and corresponding non-tumor normal colorectal tissues. Representative image of NCAPD3 staining were shown here. Scale bars: 100X = 400 μm; 400X = 100 μm. N represents Normal, T represents Tumor. Repetitions: *n* = 3 in **A**, **D**, **F**. Results were shown as mean ± s.d., *P < 0.05, **P < 0.01, ***P < 0.001, based on Student’s *t* test

RNA-seq was carried out with human CRC tissues and their corresponding normal tissues, and data analysis revealed that expression of NCAPD3 was significantly higher in tumor tissues by compared to their normal counterparts (Fig. 1A). Next, analysis of omics data from TCGA database were performed by using online bioinformatics tool GEPIA (http://gepia.cancer-pku.cn/index.html) or Assistant for Clinical Bioinformatics (https://www.aclbi.com/). Although there were no statistically significant differences in NCAPD3 expression between patients at different stages of CRC, it’s also identified that NCAPD3 was markedly overexpressed in tumor tissues by compared to normal tissues (Fig. 1B, C). Furthermore, Western blot assays showed that the protein levels of NCAPD3 were dramatically increased in CRC cell lines by compared to human normal colorectal mucosal cell line FHC (Fig. 1D upper panel). In clinical specimen, mRNA and protein levels of NCAPD3 detected by qRT-PCR, Western blot and immunochemistry analysis were also obviously higher in CRC tissues than those in normal tissues (Fig. 1D lower panel, 1E, 1F).

These results demonstrated that NCAPD3 was overexpressed in CRC, suggesting a closely relationship between NCAPD3 and CRC.

### NCAPD3 enhanced reprogramming of glucose metabolism in CRC cells

**Fig. 2:**
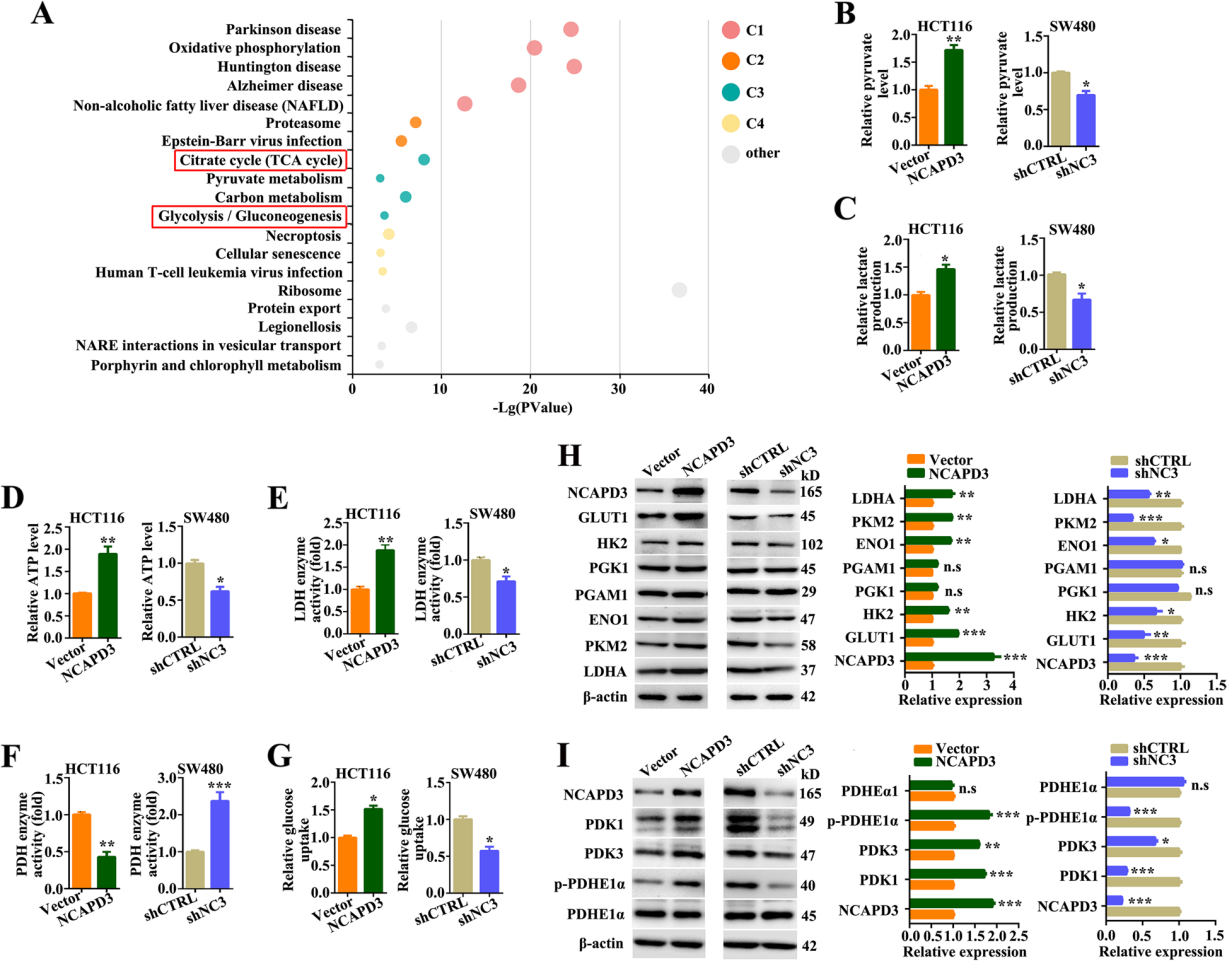
NCAPD3 enhanced glucose metabolism reprogramming in CRC cells. **A** All putative NCAPD3 interactors were functionally annotated by KOBAS online platform. Each bubble represents an enriched function, and the size of the bubble from small to large, representing different significance levels. C1, C2, C3, C4 represent different clusters. **B-G** Pyruvate level, lactate level, ATP production, lactate dehydrogenase enzyme activity, pyruvate dehydrogenase enzyme activity and glucose uptake were measurement by using related test kits in NCAPD3 overexpressed HCT116 cell line and knocked down SW480 cell line. **H, I** The protein expression of NCAPD3, GLUT1, HK2, PGK1, PGAM1, ENO1, PKM2, LDHA, PDK1, PDK3, PDHE1α and p-PDHE1α was detected by western blot in HCT116 cells with NCAPD3 overexpressed or SW480 cells with NCAPD3 knockdown. Quantitation of proteins were presented from three independent replicates. shCTRL represents shcontrol, shNC3 represents shNCAPD3. Each experiment was performed at least triplicate and results were presented as mean ± s.d., Student’s *t*-test was used to analyze the data (*P < 0.05, **P < 0.01, ***P < 0.001)

In a previous study, Ward JR and cooperators immunoprecipitated NCAPD3 from HT-29 (colon epithelial adenocarcinoma cell) and analyzed co-precipitated proteins by Liquid Chromatography-Mass Spectrometry [27]. Here, we further performed functional enrichment analysis of the published putative interactors of NCAPD3 in this reference by using the KOBAS online platform (http://kobas.cbi.pku.edu.cn/) and then revealed that NCAPD3 interactors played a role in glycolysis and TCA cycle (Fig. 2A). Based on this, we sought to figure out whether NCAPD3 modulated the glycolytic phenotype in CRC cells. From our experimental data, we found that NCAPD3 overexpression in HCT116 cells significantly increased the levels of pyruvate/lactate/ATP production, the activities of lactate dehydrogenase, and the 2-NBDG uptake, but decreased the activities of pyruvate dehydrogenase by comparing with negative controls, while the results were significantly reversed in SW480 cells with NCAPD3 knockdown (Fig. 2B-G). Again, the levels of pyruvate, lactate and ATP production could be rescued by NCAPD3 re-expression in NCAPD3-stable knockdown cells (Supplementary Fig. S1A). In addition, NCAPD3 overexpression up-regulated the levels of GLUT1, HK2, ENO1, PKM2 and LDHA proteins, but not phosphoglycerate kinase 1 (PGK1) and phosphoglycerate mutase 1 (PGAM1) proteins. As the contrast, NCAPD3 knockdown decreased the expression of these glycolytic genes except PGK1 and PGAM1 (Fig. 2H).

Pyruvate dehydrogenase E1 alpha (PDHE1α), a component of the pyruvate dehydrogenase complex which converts pyruvate to acetyl-CoA, is a key rate-limiting enzyme that determines the metabolic fate between glycolysis and TCA cycle [28]. Pyruvate dehydrogenase kinases (PDKs) phosphorylate PDHE1α at Ser293 site leading to its inactivation [29]. Hence, the levels of PDHE1α, PDKs and p-PDHE1α (Ser293) were checked in NCAPD3-knockdown SW480 cells and NCAPD3-overexpressed HCT116 cells. As shown in Fig. 2I, NCAPD3 knockdown significantly decreased the levels of PDK1, PDK3 and p-PDHE1α, and the reversed results were obtained in NCAPD3-overexpression cells, while the total protein level of PDHE1α remained unchanged.

These results demonstrated that NCAPD3 enhanced aerobic glycolysis and reduced the flux of glucose-derived carbon to the TCA cycle in CRC cells.

### NCAPD3 promoted aerobic glycolysis by regulating c-Myc and its downstream metabolism-related genes in CRC cells

**Fig. 3:**
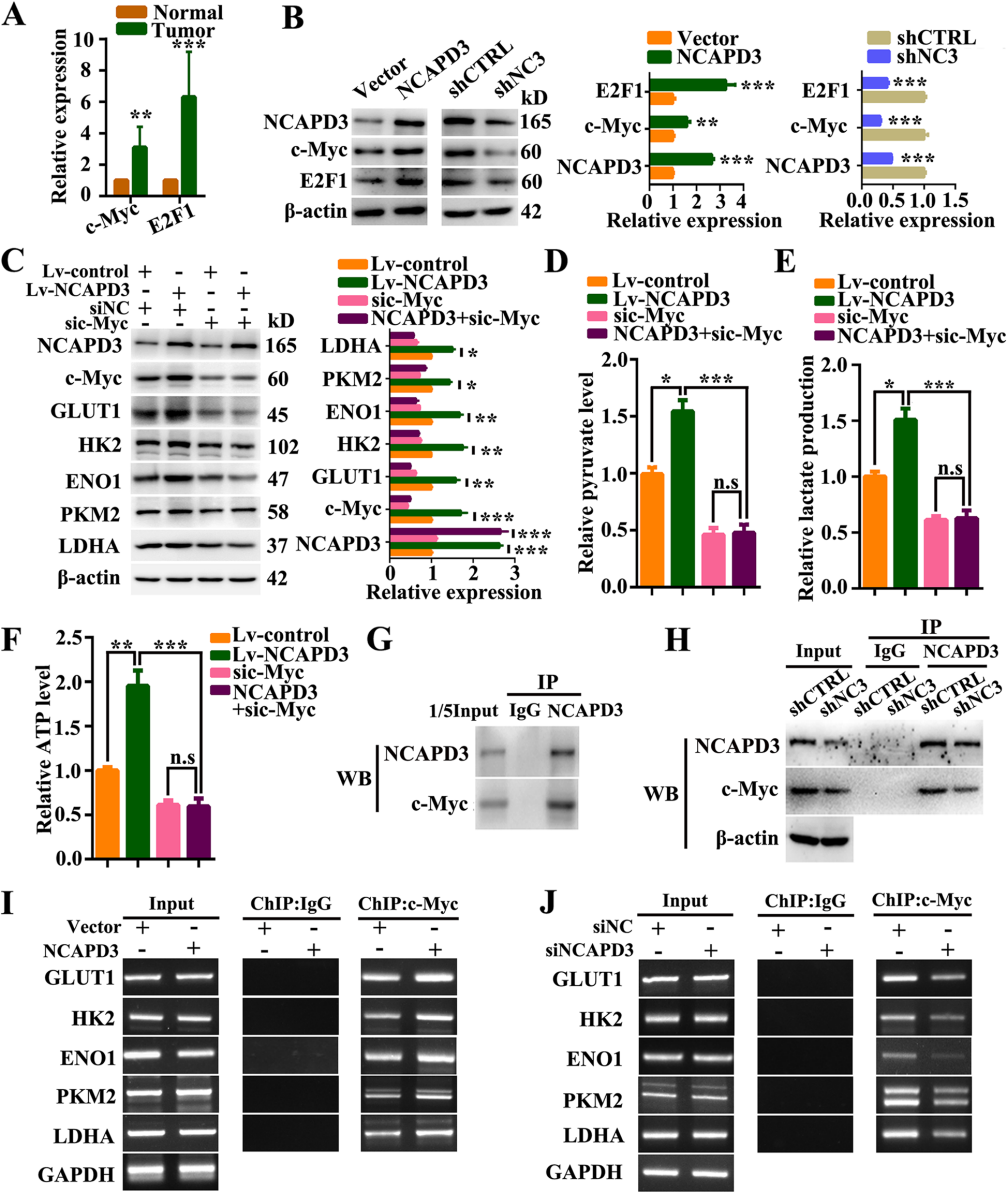
NCAPD3 upregulated c-Myc and promoted the expression of its downstream metabolic genes. **A** Relative expressions of c-Myc and E2F1 in CRC tissues and corresponding non-tumor normal tissues by analyzing our RNA-seq data of clinic specimen. **B** The protein expression of NCAPD3, c-Myc and E2F1 was detected by western blot in HCT116 cells with NCAPD3 overexpressed or SW480 cells with NCAPD3 knockdown. **C** Western blots of indicated proteins in HCT116 cells treated with indicated transfections. Quantitation of proteins were presented from three independent replicates. **D-F** Pyruvate level, lactate level, ATP production were measured with test kits in HCT116 cells treated with different treatments as indicated. **G, H** Co-immunoprecipitation (Co-IP) identified the interaction between NCAPD3 and c-Myc. **I, J** ChIP-PCR analysis of c-Myc binding at the promoter of GLUT1, HK2, ENO1, PKM2 and LDHA gene loci in NCAPD3-overexpressed HCT116 cells or NCAPD3-knockdown SW480 cells, as indicated. shCTRL represents shcontrol, shNC3 represents shNCAPD3. Each experiment was performed at least triplicate and results were shown as mean ± s.d., *P < 0.05, **P < 0.01, ***P < 0.001, based on Student’s *t*-test or two-way ANOVA

c-Myc has been reported as a key regulator of glucose metabolism by directly binding to the promoters of GLUT1, HK2, ENO1, PKM2 and LDHA genes [20]. To identify c-Myc was involved in NCAPD3-regulated aerobic glycolysis, we firstly analyzed our RNA-Seq data of clinical specimen. From the analysis, we found that the level of transcription factor c-Myc was obviously higher in CRC tissues than that in normal colorectal tissues (Fig. 3A). Based on this, we further checked the relationship between c-Myc and NCAPD3 in CRC cells using Western blotting and qRT-PCR assays. As shown in Fig. 3B and supplementary Fig. S2A, the protein and mRNA levels of c-Myc were significantly increased in HCT116 cells with overexpression of NCAPD3, while the change was reversed in SW480 cells with knockdown of NCAPD3. Moreover, the levels of c-Myc and its downstream glycolysis-related genes (GLUT1, HK2, ENO1, PKM2 and LDHA) could be rescued by NCAPD3 re-expression in NCAPD3-stable knockdown SW480 cells (Supplementary Fig. S1B).

To verify that c-Myc was necessary for the glycolysis promotion role of NCAPD3, siRNA and inhibitor of c-Myc were used in NCAPD3-stable overexpression HCT116 cells. Data showed that c-Myc knockdown and 10,058-F4 (c-Myc inhibitor) treatment reversed the high expression of GLUT1, HK2, ENO1, PKM2 and LDHA induced by NCAPD3 (Fig. 3C and Supplementary Fig. S3). This result was strengthened by the findings from pyruvate, lactate release and ATP production analyses (Fig. 3D-F), which indicated that silence or inhibition of c-Myc abolished the function of NCAPD3 in glycolysis regulation.

In addition, immunoprecipitation and western-blot assays were performed to explore the potential interaction between NCAPD3 and c-Myc in nucleus. From the data, we found that c-Myc could be co-precipitated by anti-NCAPD3 antibody in HCT116 cells. Moreover, their interaction was additionally supported by the finding from SW480 cells with NCAPD3 knockdown (Fig. 3G, H). Chromatin immunoprecipitation (ChIP)-PCR detection further confirmed that overexpression of NCAPD3 in HCT116 cells drastically increased the binding enrichment of c-Myc at the promoters of GLUT1, HK2, ENO1, PKM2 and LDHA genes, while the results were reversed in NCAPD3 knockdown SW480 cells (Fig. 3I, J).

These results demonstrated that c-Myc was involved in the glycolysis promotion role of NCAPD3 in CRC cells. NCAPD3 promoted Warburg effect by increasing the expression of c-Myc and recruiting more c-Myc to the promoter of glycolytic genes including GLUT1, HK2, ENO1, PKM2 and LDHA.

### NCAPD3 weakened TCA cycle flux in CRC cells via E2F1-mediated inhibition of pyruvate dehydrogenase

**Fig. 4:**
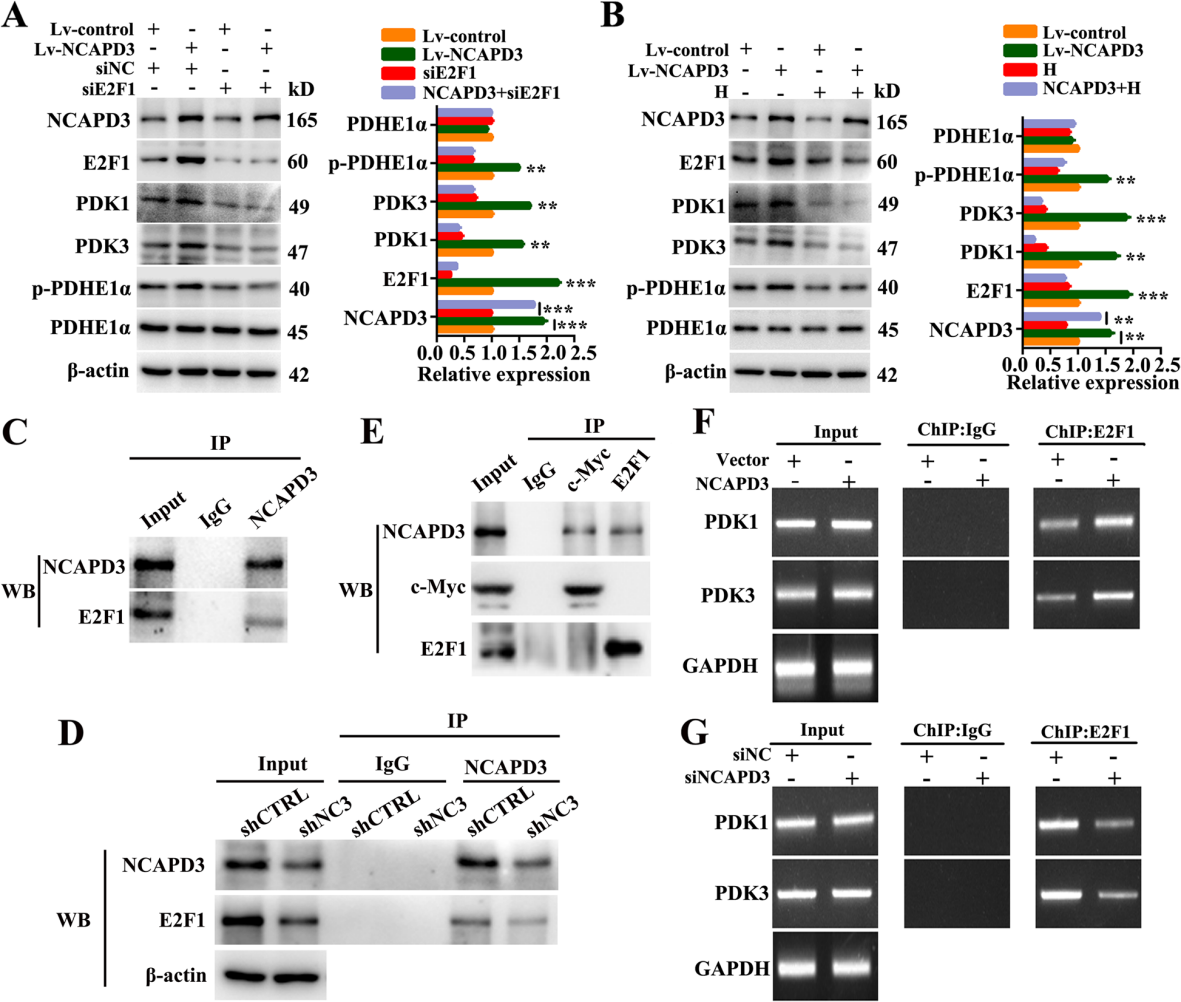
NCAPD3 inhibited TCA cycle flux via regulating the glucose metabolism switcher E2F1. **A, B** Western blot analysis of the expression of indicated proteins in CRC cells with different transfections or E2F1 inhibitor HLM006474 treatment. Quantitation of proteins were presented from three independent replicates. **C, D** Immunoprecipitation identified the interaction between NCAPD3 and E2F1. **E** The reciprocal IP was performed with c-Myc or E2F1 antibody followed by WB detection of NCAPD3, c-Myc and E2F1. **F, G** ChIP-PCR analysis of E2F1 binding at the promoter of PDK1 and PDK3 gene loci in cells with indicated transfection. **H** represents HLM006474 (E2F1 inhibitor). shCTRL represents shcontrol, shNC3 represents shNCAPD3. Each experiment was performed at least triplicate and results were shown as mean ± s.d., *P < 0.05, **P < 0.01, ***P < 0.001, based on Student’s *t*-test or two-way ANOVA

Previous studies demonstrated that E2F1 can effectively induce a metabolic switch from glucose oxidation to aerobic glycolysis [30]. All pyruvate dehydrogenase kinase (PDK) family members harbor E2F1 binding sites in their promoter regions [25]. Therefore, we thought that NCAPD3 weakened the TCA cycle flux in CRC cells via glucose metabolism switcher E2F1. Based on this, we firstly analyzed our RNA-Seq data of clinical specimen and found that level of transcription factor E2F1 was higher in CRC tissues than that in normal tissues (Fig. 3A). And then, western-blot and qRT-PCR data further demonstrated that NCAPD3 could positively regulate E2F1 expression in CRC cells (Fig. 3B and Supplementary Fig. S2A). In addition, siRNA silencing E2F1 or treatment with E2F1 inhibitor HLM006474 in NCAPD3-stable overexpression HCT116 cells were carried out to identify the importance of E2F1 in NCAPD3-mediated inhibition of TCA cycle. As shown in Fig. 4A and B, NCAPD3 overexpression upregulated the levels of E2F1 and its target PDK1 and PDK3 genes and then resulted in the increase of p-PDHE1α (but the total PDHE1α) and suppression of PDH activity, while knockdown or inhibition of E2F1 with siRNA or inhibitor reversed the results induced by NCAPD3 overexpression. Moreover, immunoprecipitation assays showed that E2F1 could be co-precipitated by anti-NCAPD3 antibody in HCT116 cells or SW480 cells with NCAPD3 knockdown (Fig. 4C, D), suggesting that NCAPD3 and E2F1 interacted in CRC cells. Reciprocal IP with anti-c-Myc antibody or anti-E2F1 antibody in HCT116 cells were carried to detect the interaction between them. Our data showed that c-Myc could not be co-precipitated by anti-E2F1 antibody in HCT116 cells, and vice versa, suggesting that NCAPD3, c-Myc and E2F1 could not form a trimeric complex (Fig. 4E).

To determine E2F1 promoting the expression of PDK1 and PDK3, we conducted ChIP-PCR assay. Results showed that NCAPD3 overexpression increased the binding enrichment of E2F1 to the gene promoters of PDK1 and PDK3 in HCT116 cells, and the reversed results were obtained in NCAPD3 knockdown SW480 cells (Fig. 4F, G). Moreover, the levels of E2F1, PDK1 and PDK3 could be rescued by NCAPD3 re-expression (Supplementary Fig. S1C).

These data demonstrated that NCAPD3 weakened TCA cycle flux by increasing the expression of E2F1, interacting with E2F1 and then recruiting more E2F1 to the promoters of PDK1 and PDK3, resulting in the increase of PDK1 and PDK3 levels, which finally increased PDHE1α phosphorylation and inactivated PDH complex.

### NCAPD3 functioned as an enhancer in contributing to the proliferation and metastasis of CRC cells

**Fig. 5:**
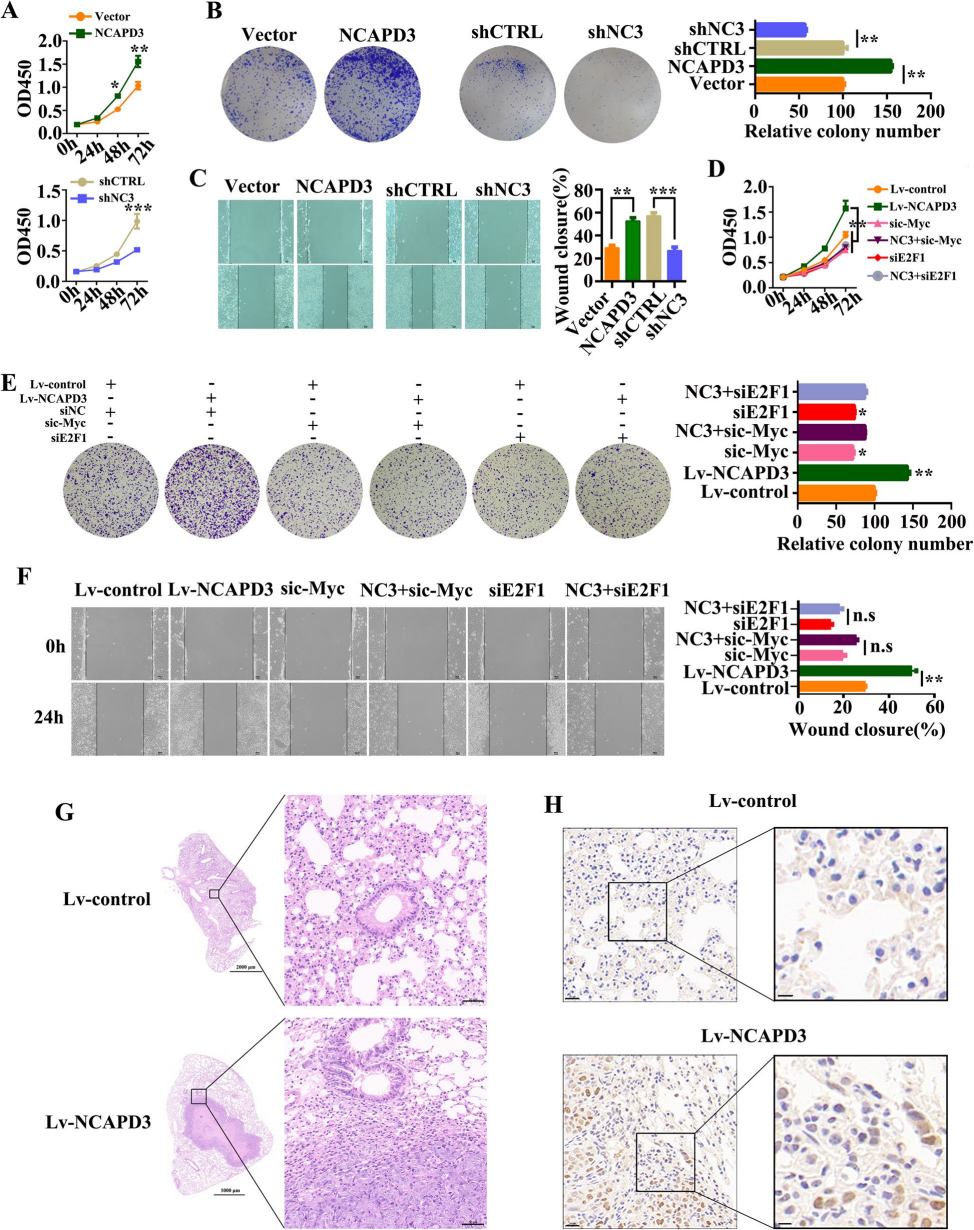
NCAPD3 promoted CRC cell proliferation, migration and tumor metastasis. **A-C** Cell viability, colony formation and migration were measured by CCK8 assay, colony formation assay and wound-healing assay after NCAPD3 overexpression or knockdown. Scale bar: 50 μm. **D-F** CCK-8 assay, colony formation analysis and wound-healing assay were performed under different transfections as indicated in figures. Scale bar: 50 μm. **G** Representative images of HE staining of lung tissues in NCAPD3-overexpressed and control group, *n* = 5. Scale bar: 50 μm. **H** Representative Ki67 IHC staining of lung tissues from mice, *n* = 5. Scale bar: 20 μm. NC3 represents NCAPD3, shCTRL represents shcontrol, shNC3 represents shNCAPD3, siNC represents negative control siRNA. All experiments were implemented three times. Student’s *t*-test was used to analyze the data (*P < 0.05, **P < 0.01, ***P < 0.001)

Warburg effect in cancer cells is always advantage to promote cell growth, proliferation and metastasis [31]. To identify this function of NCAPD3-induced Warburg effect, CCK-8 assay and colony formation assay were firstly carried out. From our data, NCAPD3 overexpression promoted HCT116 cell proliferation and colony formation (Fig. 5A, B). Furthermore, scratch wound healing assay data showed that ectopic expression of NCAPD3 significantly increased the migration of HCT116 cells by comparing with the controls (Fig. 5C). In NCAPD3-knockdown SW480 cells, cell proliferation, colony formation and migration were all greatly weakened (Fig. 5A-C), while the weakened results could be mainly rescued by NCAPD3 re-expression (Supplementary Fig. S4A-C). In addition, NCAPD3-enhanced cell proliferation, colony formation and migration could be suppressed by siRNA knockdown (Fig. 5D-F) and inhibitor inhibition (Supplementary Fig. S4D) of c-Myc or E2F1. It’s reported that reprogramming of glucose metabolism, especially glycolysis, is profoundly implicated in tumor metastasis [32]. To identify the role of NCAPD3 in affecting CRC metastasis, in vivo metastasis experiment was carried out by injecting NCAPD3-stable transfection HCT116 cells and control cells in the tail vein of nude mice. As shown in Fig. 5G, histological analysis revealed that mice receiving the HCT116-NCAPD3 subline formed lung metastatic foci by compared with control group. Consistently, overexpression of NCAPD3 resulted in a stronger Ki67 staining in lung metastatic tumors in comparison to the control group (Fig. 5H and Supplementary Fig. S4E).

These results indicated that c-Myc and E2F1 involved in NCAPD3 promoting CRC cell proliferation and migration in vitro. In addition, in vivo data also demonstrated that overexpression of NCAPD3 in HCT116 cells promoted tumor lung metastasis.

### NCAPD3 promoted tumor growth by enhancing CRC cell aerobic glycolysis in the subcutaneous xenograft mouse model

**Fig. 6:**
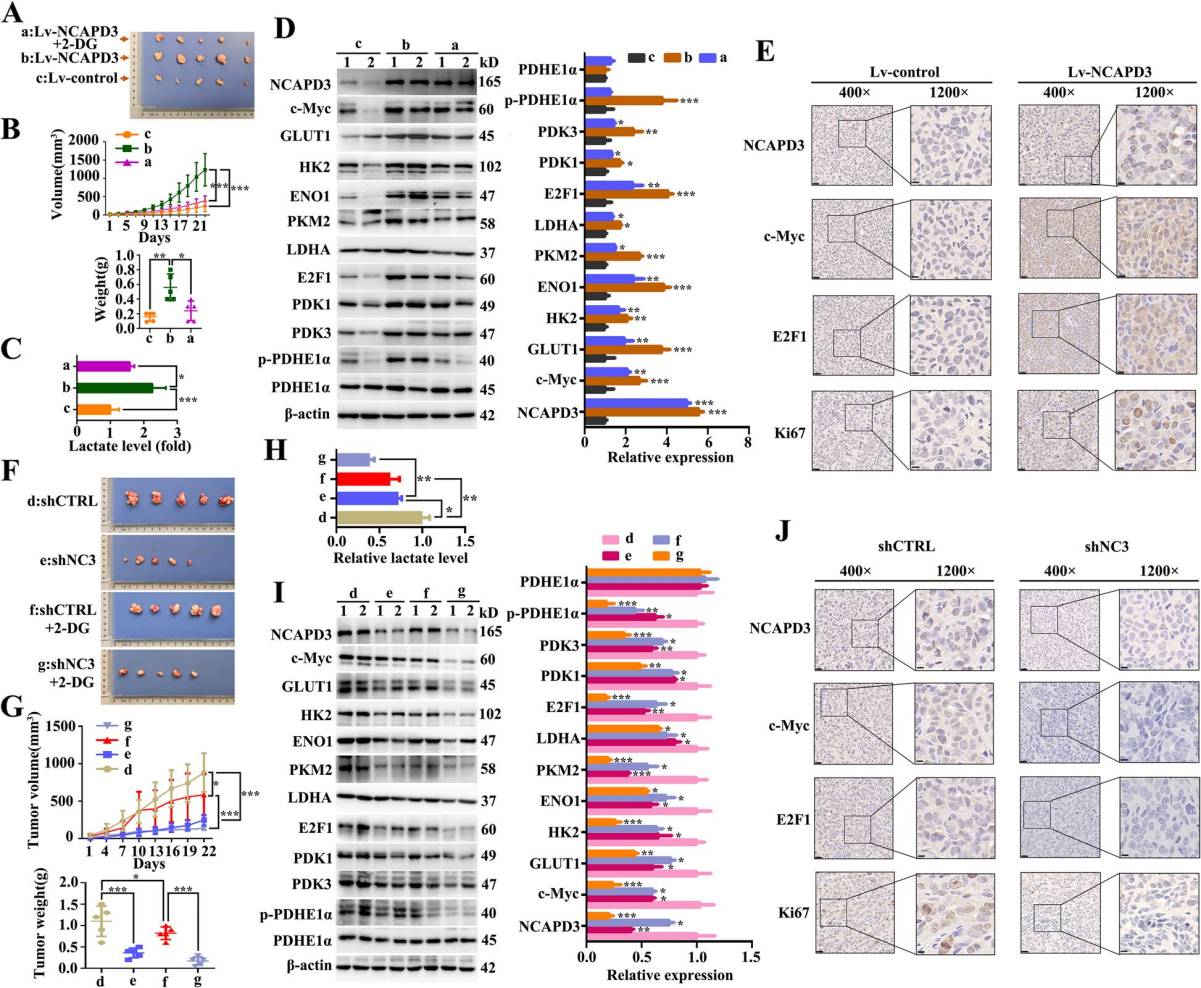
NCAPD3 enhanced tumor growth and aerobic glycolysis in a xenograft tumor mice model. **A, F** Tumor images dissected from the nude mice after being subcutaneously injected with HCT116 cells or SW480 cells, followed by treatment with intraperitoneal injection of PBS or 2-DG. **a** represents Lv-NCAPD3 + 2-DG group, **b** represents Lv-NCAPD3 group, **c** represents Lv-control group, **d** represents shcontrol group, **e** represents shNCAPD3 group, **f** represents shcontrol + 2-DG group, **g** represents shNCAPD3 + 2-DG group, *n* = 5. Scale bar: 1 cm. **B, G** Average tumor volume and weight of each mice group were measured and presented. **C, H** Relative lactate levels in xenograft tumor with indicated treatment were tested and presented. **D, I** Expression of proteins related to glycolysis and TCA cycle were detected in xenografts after indicated treatment by Western blot. 1, 2 represent tumor tissues from different mice. **E, J** Representative images of IHC staining of NCAPD3, c-Myc, E2F1 and Ki-67 on tumor sections. Scale bar: 20 μm. shCTRL represents shcontrol, shNC3 represents shNCAPD3. Statistical results were shown as mean ± s.d., *P < 0.05, **P < 0.01, ***P < 0.001, based on two-way ANOVA or Student’s *t*-test

The in vivo experiments were conducted using subcutaneous xenograft mouse model, generated by subcutaneous injection of NCAPD3-stable overexpression HCT116 cells or NCAPD3-stable knockdown SW480 cells and their respective control cells. From the experimental data, we found that the size and weight of xenograft tumors of NCAPD3-overexpression group were evidently larger when compared to control group (Fig. 6A, B), while the tumor size and weight were significant decreased in NCAPD3-overexpression following treatment with 2-DG group as compared to NCAPD3-overexpression group (Fig. 6A, B). The lactate level in xenograft tumors formed by HCT116-NCAPD3 cells was significantly increased in comparison to the tumors formed by control cells, while the lactate level of the tumors formed by HCT116-NCAPD3 cells following treatment with 2-DG was decreased when compared to the tumors formed just by HCT116-NCAPD3 cells (Fig. 6C). The effect of NCAPD3 overexpression on the glycolysis-related protein levels in the xenograft tumors were consistent with the conclusion derived from lactate release assay (Fig. 6D). Furthermore, NCAPD3 up-regulated the mRNA expressions of c-Myc and E2F1 in the xenograft tumors (Supplementary Fig. S2B). These results indicated that NCAPD3 promoted tumor growth and Warburg effect, which could be partially eliminated by 2-DG treatment.

The results of xenograft mouse formed by stable NCAPD3-knockdown SW480 cells demonstrated that NCAPD3 depletion significantly inhibited tumor growth and the levels of lactate release and glycolysis-related proteins were reduced; and these effects of NCAPD3-knockdown were further strengthened by extra treatment with 2-DG. (Fig. 6F-I). NCAPD3 down-regulated the mRNA expressions of c-Myc and E2F1 in the xenograft tumors (Supplementary Fig. S2B). Moreover, immunohistochemistry results showed a stronger staining of c-Myc, E2F1 and Ki67 in the xenograft tumors formed by NCAPD3-stable overexpression HCT116 cells, whereas it’s reversed in the xenograft tumors formed by NCAPD3-stable knockdown SW480 cells as compared to the controls (Fig. 6E, J).

These in vivo results, consistently with findings from in vitro assays, indicated that NCAPD3 promoted CRC growth via enhancing the Warburg effect of CRC cells.

### NCAPD3 knockout attenuated colorectal carcinogenesis in AOM/DSS-induced mouse model

**Fig. 7:**
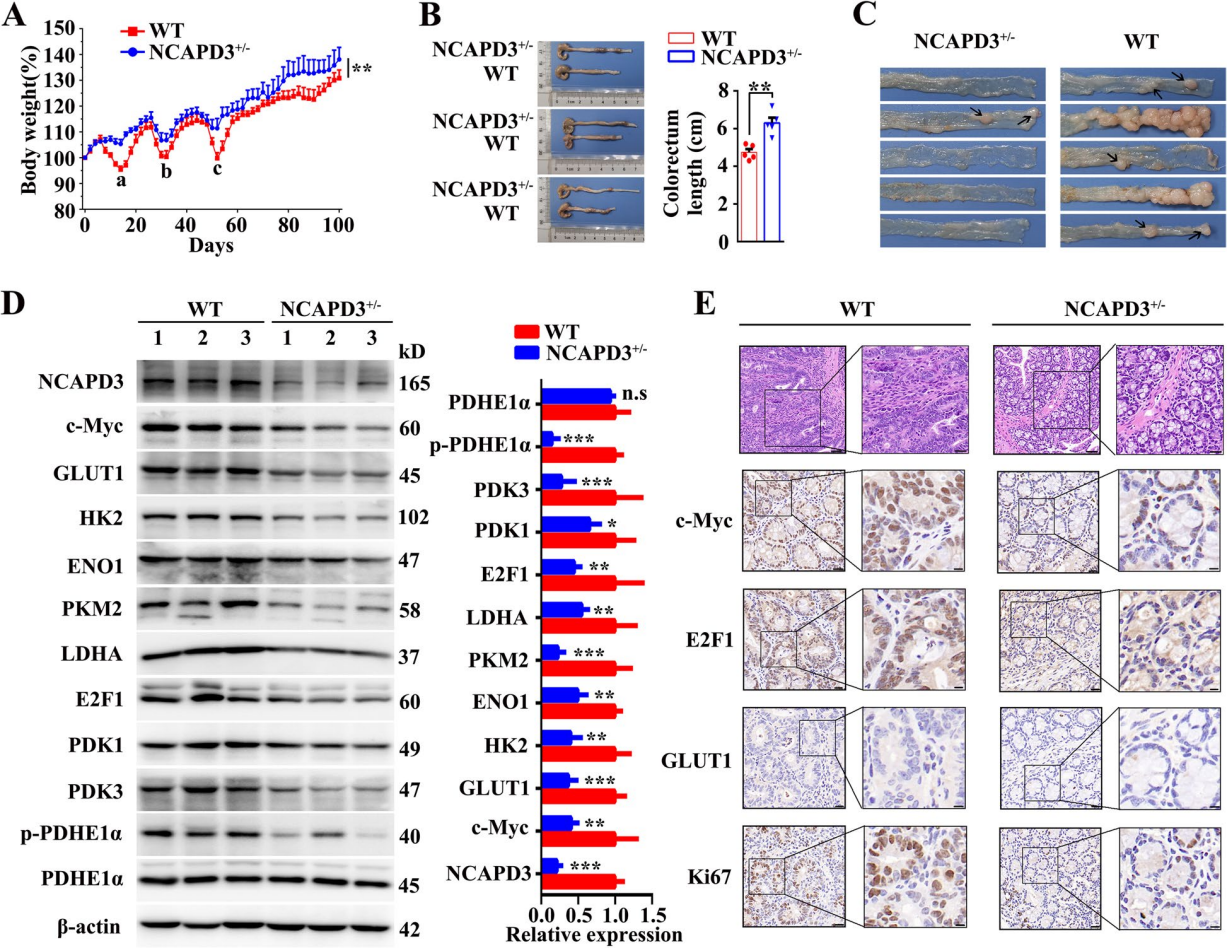
Knockout of NCAPD3 attenuated colorectal carcinogenesis in AOM/DSS-induced mice model. **A** Body weight changes of wild type (WT) and NCAPD3^±^ mice during AOM/DSS treatment (*n* = 5). **B** Typical intestines of mice on day 108 were dissected and photographed after mice were euthanized. Scale bar: 1 cm. Colorectal lengths of WT and NCAPD3^±^ mice were measured (*n* = 5). **C** Representative images of colorectal tumors from WT and NCAPD3^±^ mice (*n* = 5). **D** Expression of proteins related to glycolysis and TCA cycle were detected in tumor sections from WT and NCAPD3^±^ mice by Western blot. 1, 2, 3 represent tumor tissues from different mice **E** Histological analysis and representative IHC images of c-Myc, E2F1, GLUT1, Ki67 on the tumor sections from WT and NCAPD3.^±^ mice. Scale bar: 20 μm. Results were shown as mean ± s.d., *P < 0.05, **P < 0.01, ***P < 0.001, based on Student’s *t*-test or two-way ANOVA

To confirm the effect of NCAPD3 on tumorigenesis, *NCAPD3* knockout mice (NCAPD3^±^ mice) was generated and identified by PCR analysis (Supplementary Fig. S5A, B); and the mouse model of colitis-associated colorectal cancer was established by intraperitoneally injecting with 10 mg/kg AOM and following by three cycles of 2% DSS drinking treatment (Supplementary Fig. S5C). After induction of colorectal tumorigenesis, mice were euthanized on day 108 and the intestines were collected for analysis. By comparing with wild type (WT) mice, NCAPD3^±^ mice exhibited less body weight loss during three times of DSS treatment (a, b and c sites of Fig. 7A) and had a higher body weight and the longer length of colorectum at the end of experiment (Fig. 7A, B), indicating that NCAPD3-deficient mice were less susceptibility to DSS-induced colitis than WT mice. Further, it’s found that most tumors located at the distal region of the intestine; and both tumor number and tumor size in NCAPD3^±^ mice were significantly lower than those in WT littermates (Fig. 7C). Western blotting data confirmed that *NCAPD3* knockout led to significant downregulation of c-Myc, E2F1 and their downstream target genes at the protein level, whereas total PDHE1α protein expression remain unchanged (Fig. 7D). Moreover, *NCAPD3* knockout down-regulated the mRNA expressions of c-Myc and E2F1 (Supplementary Fig. S2C). Histologically, WT mice presented a loss of epithelial integrity and nearly complete loss of crypts by compared with NCAPD3^±^ mice (Fig. 7E). In addition, IHC analysis also showed that *NCAPD3* knockout led to remarkably weaker staining of c-Myc, E2F1 and GLUT1 (Fig. 7E), as well as a significant decrease of the proliferation rates of colorectal tumors in NCAPD3^±^ mice, as evidenced by lower proportions of Ki-67 nuclear staining (Fig. 7E).

These in vivo results revealed that NCAPD3 deficiency attenuated colorectal tumorigenesis in AOM/DSS induced mouse model via suppressing the glucose metabolism reprogramming and glycolysis.

### Clinical significance of NCAPD3 and its correlation with metabolism-related genes in CRC patients

**Fig. 8:**
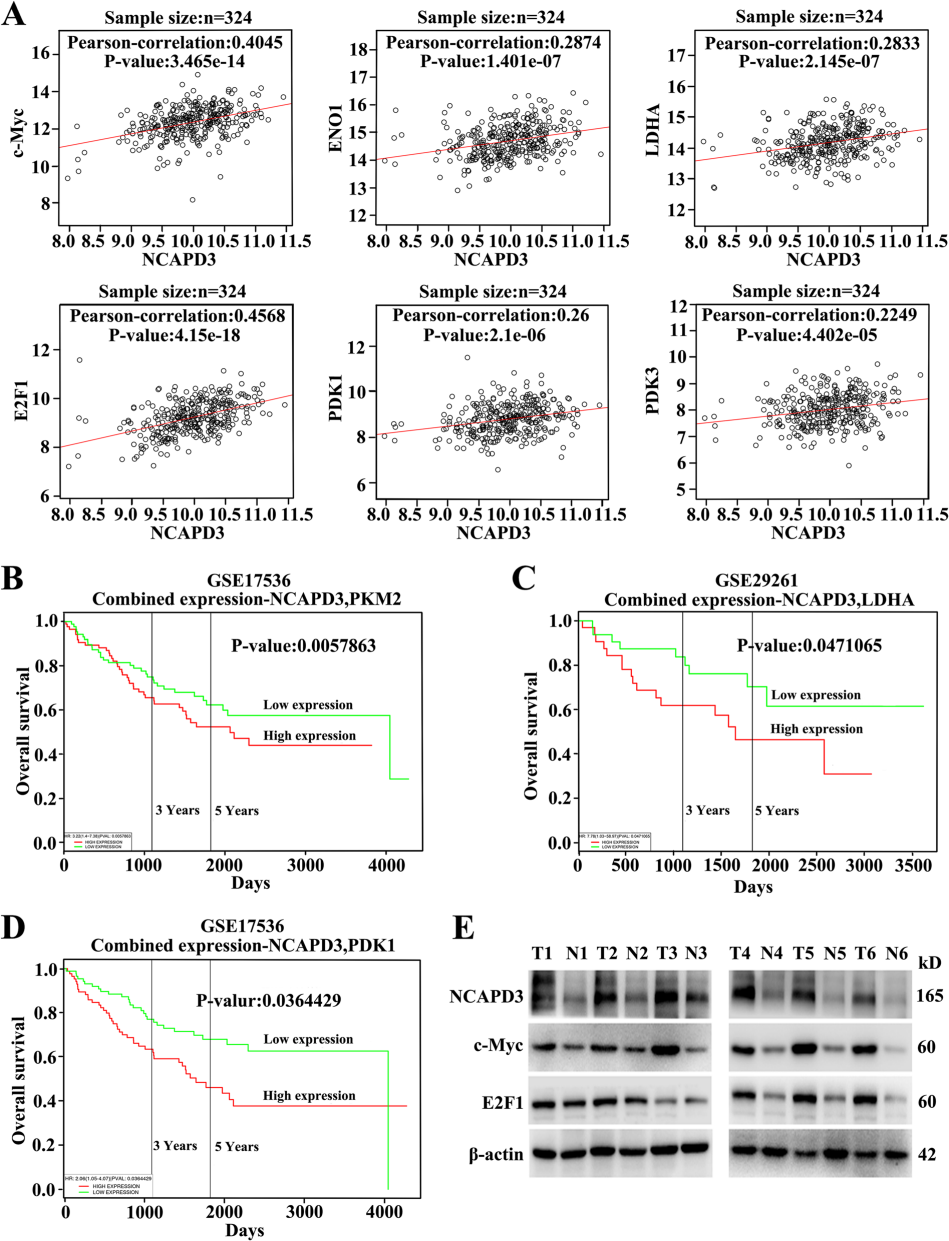
Clinical significance of NCAPD3 and its correlation with metabolism-related genes in CRC patients. **A** Pearson analysis of the correlation between the levels of NCAPD3 and c-Myc, ENO1, LDHA, E2F1, PDK1, PDK3 expression in the TCGA cohort. **B-D** Kaplane-Meier survival analysis by combination of NCAPD3 with PKM2 or LDHA or PDK1 in the TCGA cohort of CRC patients. **E** Western blot analysis for NCAPD3, c-Myc and E2F1 expression in clinic specimen (*n* = 6). N represents Normal, T represents Tumor

To further confirm the above results and investigate the clinical significance of NCAPD3, we analyzed the clinical data from TCGA dataset by using Linkedomics platform (http://www.linkedomics.org). From the analysis, we found that NCAPD3 showed a strongly positive correlation with c-Myc, ENO1, LDHA, E2F1, PDK1 and PDK3 in CRC tissues respectively, which further supported our in vitro and in vivo experimental findings (Fig. 8A). Moreover, the Kaplan–Meier survival plots of CRC patients were appraised by PROGgeneV2 platform (http://genomics.jefferson.edu/proggene/) using the joint expression status of NCAPD3 with PKM2, LDHA and PDK1 respectively. As illustrated in Fig. 8B-D, patients with NCAPD3-High/PKM2-High, NCAPD3-High/LDHA-High and NCAPD3-High/PDK1-High exhibited shorter overall survival. Finally, we assessed the expression levels of NCAPD3, c-Myc and E2F1 in 6 pairs of clinical CRC tissues and their corresponding adjacent normal tissues using Western blot assay. By comparing with the adjacent normal tissues, CRC tissues had higher NCAPD3, c-Myc and E2F1 expression (Fig. 8E).

These results indicated that NCAPD3 and its induced Warburg effect might play a critical role in colorectal tumorigenesis and CRC progression, suggesting a potential CRC marker and clinical treatment target.

## Discussion

Condensin complexes have pivotal functions in regulating chromosome condensation and segregation during cell cycles [33]. It has been suggested in the literature that subunits of condensing I and condensin II are associated with tumorigenesis [34]. Recently, studies have reported that NCAPD3 localizes to the exterior surface of mitochondria and affects mitochondrial oxidative stress responses [35]. The research on NCAPD3 mainly focuses on its role in condensation and segregation during cell division, little is known about its bio-functions and molecular mechanisms in tumors. This study presented the evidence that NCAPD3 expression was upregulated in CRC. In vitro and in vivo experiments clearly demonstrated that NCAPD3 promoted colorectal tumorigenesis, CRC cell proliferation, migration and metastasis, suggesting that NCAPD3 was an oncogene and played a carcinogenic potential role in CRC.

Glucose metabolism reprogramming is an important feature of tumor cells. Large amount of evidence has emerged in supporting the critical roles of glycolytic metabolism in promoting tumorigenesis and progression in various cancer types [36]. Although it has been recognized that glycolysis is highly active (Warburg effect) and plays an important role in cell carcinogenesis and cancer development, the molecular mechanism by which glycolysis is highly activated is not well- characterized. We elucidated for the first time that NCAPD3 was involved in glucose metabolism reprogramming process and induced a metabolic switch from glucose oxidation to aerobic glycolysis, as well as enhanced the Warburg effect in CRC cells. Further, it’s figured out that NCAPD3 switched the glucose metabolism and enhanced the glucose glycolysis via up-regulating the activities of c-Myc and E2F1 pathways, which finally resulted in the colon tumorigenesis and CRC progression (Supplementary Fig. S6).

It’s reported that c-Myc played an important function in enhancing the glycolytic metabolism of cancer cells, which promoted the progression and metastasis of types of cancers in clinic, including CRC [37]. As a transcription factor, c-Myc activates many genes that are involved in various cellular processes, including glucose metabolism process, especially glycolysis [38]. In many types of cancers, c-Myc is always activated and plays key role in reprogramming glycolytic metabolism by activating its target genes, like GLUT1, HK2, PKM2 and LDHA, and then results in cancer cell proliferation, migration and metastasis [39, 40]. In CRC cells, lncRNA GLCC1 direct interaction with HSP90 to protected c-Myc from ubiquitination and further specified the transcriptional modification pattern of c-Myc target genes such as LDHA, consequently reprogramming glycolytic metabolism of cells [40]. In glioblastoma, mTOR complex 2 (mTORC2) up-regulates the level of c-Myc and activates c-Myc by increasing the acetylation levels of FoxO1 and FoxO3 and then promotes glycolytic metabolism [41]. In many types of cancers, c-Myc can directly transcriptionally activate glycolytic genes, thereby promoting the activity of glycolysis with increased glucose uptake and fast conversion of glucose to lactate [42]. Lots of studies show that c-Myc activation in cancer cells is a key in promoting Warburg phenomenon. In this study, we found that NCAPD3 was higher expression in CRC and directly proportional relationship with c-Myc. NCAPD3 higher expression in CRC cells could up-regulate the expression level of c-Myc and recruit more c-Myc to its downstream target glycolytic genes, such as GLUT1, HK2, ENO1, PKM2 and LDHA, and finally promoted colorectal carcinogenesis and CRC cell growth, proliferation, and metastasis by enhancing the aerobic glycolysis process. As we known, NCAPD3 was a subunit of condensin II and functioned mainly as the chromatin condensation factor related to cell mitosis rather than a transcription factor. NCAPD3 increased the level of c-Myc in CRC cells was not clear and needed to be further investigated.

E2F1 has been found to be deregulated in many types of cancers [22]. Recent evidence showed that E2F1 could promote metabolic switch by both enhancing glycolysis and repressing oxidative metabolism under stressful conditions [43]. During hepatocellular carcinoma progression, E2F1 promoted the expression of its genes involved in glycolysis, which contributed to the Warburg effect [44]. In bladder and prostate cancer cell lines, E2F1 enhanced cancer cell glycolysis by directly binding to the proximal SIRT6 promoter and suppressed SIRT6 expression, as well as significantly enhanced glucose uptake and lactate production [45]. Besides enhancing the expression of glycolytic genes, E2F1 also blocked the oxidation of glucose in mitochondria by promoting the expression of PDKs. In bone marrow progenitor cells, E2F1 was a potent activator of PDK2/4 gene expression; genetic deletion of E2F1 decreased PDK2 and PDK4 expression and increased oxidative metabolism [46]. E2F1 directly regulated PDK4 levels and suppressed mitochondrial glucose oxidation in myoblasts and fibroblasts [47]. Our latest study also identified the interaction between NCAPD3 and E2F1 in prostate cancer which increased the binding of E2F1 to the promoter of EZH2 [48]. In this text, we found that NCAPD3 could up-regulate the expression levels of PDK1 and PDK3 by increasing the expression of E2F1 and recruiting E2F1 to the promoters of its target genes PDK1 and PDK3, and then increased the phosphorylation level of PDHE1α at S293 and inactivated the enzymatic activity of PDH, which finally resulted in shifting the energy production towards aerobic glycolysis instead of TCA cycle. Therefore, NCAPD3, on the other hand, could inhibit pyruvate from entering TCA cycle by enhancing the TF activity of E2F1. As to the details of E2F1 participating in NCAPD3-enhanced cell glycolysis and the detailed mechanism of NCAPD3-mediated upregulation of E2F1, they needed to be further figured out in the next.

## Conclusions

In conclusion, we identified a novel role for NCAPD3 in promoting glucose metabolic reprogramming in CRC cells. In vivo and in vitro, NCAPD3 could reprogram the glucose metabolism by enhancing glycolysis process and suppressing TCA cycle via both increasing the expressions of c-Myc and E2F1 and recruiting these two transcription factors to the promoters of their target genes, suggesting that NCAPD3 played an important role in regulating glucose metabolism for the occurrence and progression of CRC.

## Supplementary Information

**Additional file 1: Figure S1.** NCAPD3 re-expression rescued the levels of pyruvate, lactate, ATP and proteins related to glucose metabolism. **Figure S2.** The effect of NCAPD3 on c-Myc, E2F1 at the transcriptional level. **Figure S3.** 10058-F4 treatment reversed high-expression of GLUT1, HK2, ENO1, PKM2 and LDHA that induced by NCAPD3. **Figure S4.** The effect of NCAPD3 re-expression or 10058-F4/HLM006474 treatment on cell proliferation, colony formation, migration. **Figure S5.** Generation of NCAPD3+/- mice and Schematic representation of the AOM/DSS procedure. **Figure S6.** Schematic diagram of NCAPD3 functional and mechanism in CRC.

**Additional file 2: Table S1.** The sequence of siRNA oligonucleotides targeting NCAPD3, C-myc, E2F1 and negative control. **Table S2.** Detailed information of antibodies and reagents. **Table S3.** The primer sequences used for qRT-PCR assay.** Table S4.** Primer sequences used for ChIP assay.

## Data Availability

The data analyzed in this study were obtained from Gene Expression Omnibus (GEO) at GSE17536 and GSE29261. The human sequence data generated in this study were not publicly available due to patient privacy requirements but were available upon reasonable request from the corresponding author. Other data generated in this study were available within the article and its supplementary data files.

## Abbreviations

AOM: Azoxymethane
CRC: Colorectal cancer
ChIP: Chromatin immunoprecipitation
DSS: Dextran sodium sulfate
ENO1: Enolase 1
GLUT1: Glucose transporter 1
HK2: Hexokinase 2
IHC: Immunohistochemistry
LDHA: Lactate dehydrogenase A
NCAPD3: Non-SMC Condensin II Complex Subunit D3
PKM2: Pyruvate kinase M2
PGK1: Phosphoglycerate kinase 1
PGAM1: Phosphoglycerate mutase 1
PDK1: Pyruvate dehydrogenase kinase 1
PDK3: Pyruvate dehydrogenase kinase 3
PDHE1α: Pyruvate dehydrogenase E1 alpha
2-DG: 2-Deoxy-D-glucose
2-NBDG: 2-(N-(7-Nitrobenz-2-oxa-1,3-diazol-4-yl)-Amino)-2-Deoxyglucose
