# Supplementary material for: NCAPD3 enhances Warburg effect through c-myc and E2F1 and promotes the occurrence and progression of colorectal cancer
Source: J Exp Clin Cancer Res. 2022 Jun 11;41:198. doi: 10.1186/s13046-022-02412-3 (PMC9188166; doi:10.1186/s13046-022-02412-3)
Supplement: Supplementary file 1 — Additional file 1: Figure S1. NCAPD3 re-expression rescued the levels of pyruvate, lactate, ATP and proteins related to glucose metabolism. Figure S2. The effect of NCAPD3 on c-Myc, E2F1 at the transcriptional level. Figure S3. 10058-F4 treatment reversed high-expression of GLUT1, HK2, ENO1, PKM2 and LDHA that induced by NCAPD3. Figure S4. The effect of NCAPD3 re-expression or 10058-F4/HLM006474 treatment on cell proliferation, colony formation, migration. Figure S5. Generation of NCAPD3+/- mice and Schematic representation of the AOM/DSS procedure. Figure S6. Schematic diagram of NCAPD3 functional and mechanism in CRC. [file 13046_2022_2412_MOESM1_ESM.docx]

**Supplementary figures**

**Figure S1. NCAPD3 re-expression rescued the levels of pyruvate, lactate, ATP and proteins related to glucose metabolism.**


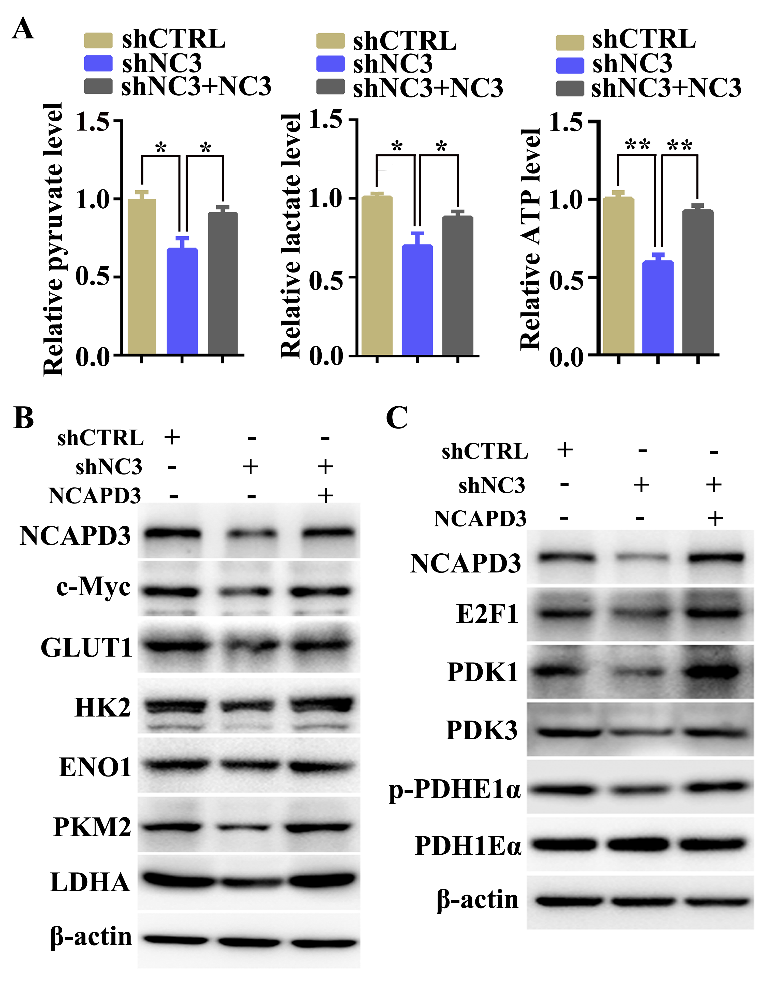


(A) Pyruvate level, lactate level, ATP production in SW480 cells with indicated treatment were measurement by using commercial kits. (B, C) Western blots of indicated proteins in SW480 cells with different treatment. Each experiment was performed at least triplicate and results are presented as mean ± s.d., Student’s *t*-test was used to analyze the data (**P* < 0.05, ***P* < 0.01). shCTRL: shcontrol; shNC3: shNCAPD3 (stable knockdown); shNC3+NC3: shNCAPD3 (stable knockdown) +NCAPD3

**Figure S2. The effect of NCAPD3 on** **c-Myc, E2F1 at the transcriptional level****.**


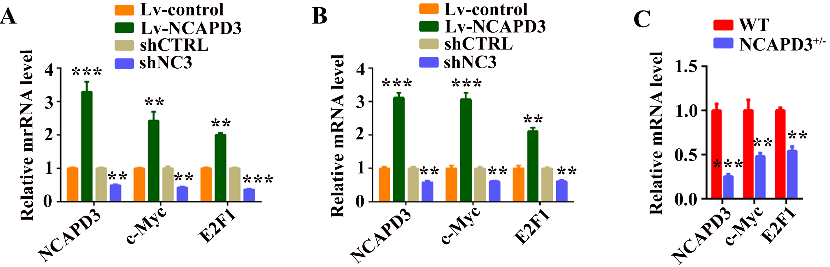


(A) Relative mRNA level of c-Myc and E2F1 in HCT116 cells with NCAPD3 overexpression or SW480 cells with NCAPD3 knockdown. (B) Relative mRNA level of c-Myc and E2F1 in mouse xenografts. (C) Relative mRNA level of c-Myc and E2F1 in NCAPD3^+/-^ mice or WT mice. shCTRL: shcontrol; shNC3: shNCAPD3. Each experiment was performed at least triplicate and results are presented as mean ± s.d., Student’s *t*-test was used to analyze the data (**P* < 0.05, ***P* < 0.01, ****P* < 0.001).

**Figure S3.** **10058-F4 treatment reversed high-expression of GLUT1, HK2, ENO1, PKM2 and LDHA that induced by NCAPD3.**


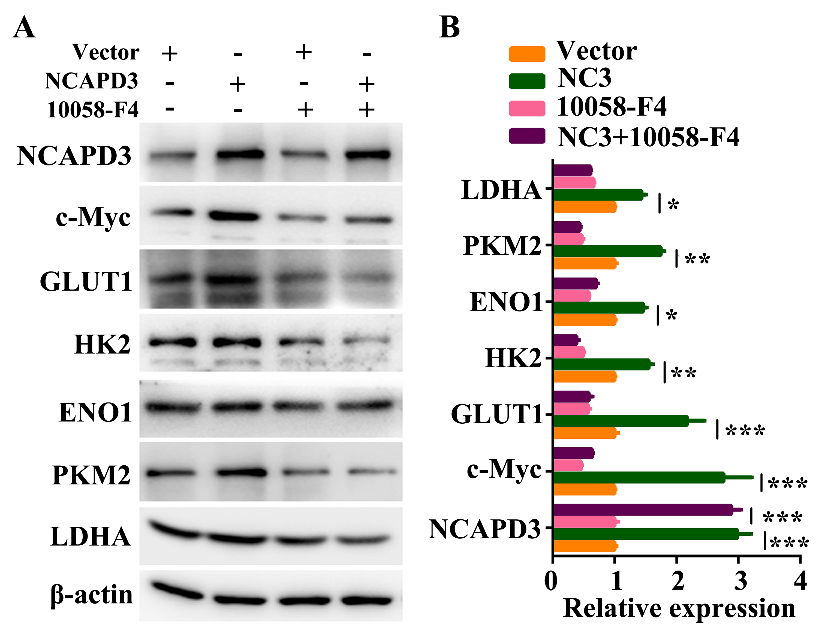


(A) Western blots of indicated proteins in HCT116 cells with different treatment and statistical results are described in (B). Repetitions = 3. NC3: NCAPD3. Results are presented as mean ± s.d., Student’s *t*-test was used to analyze the data (**P* < 0.05, ***P* < 0.01, ****P* < 0.001).

**Figure S4.** **The effect of NCAPD3 re-expression or 10058-F4 / HLM006474 treatment on cell proliferation, colony formation, migration.**


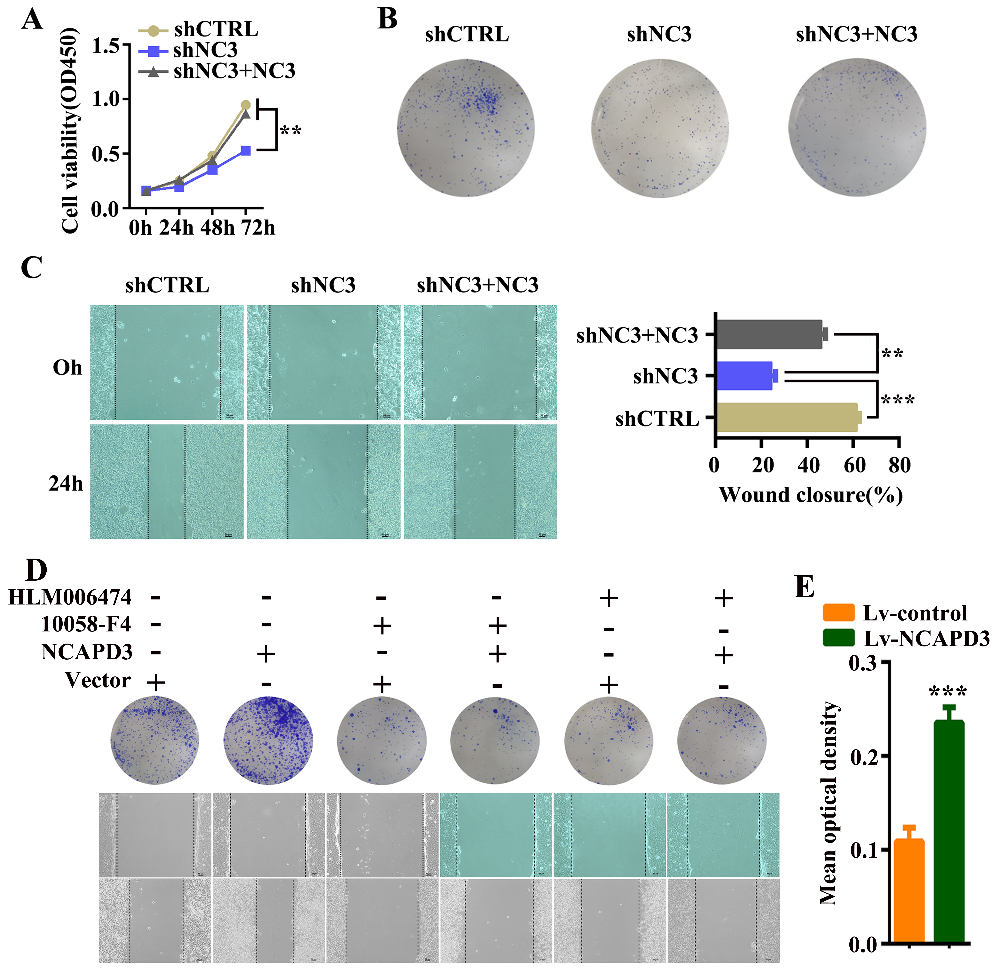


(A-C) Cell viability colony formation and migration were measured by CCK8 assay, colony formation assay and wound-healing assay. (D) Colony formation and cell migration were measured by colony formation assay and wound-healing assay in HCT116 cells with different treatment. (E) Quantification analysis of Ki67 IHC staining in the indicated lung metastatic tumors. shCTRL: shcontrol; shNC3: shNCAPD3. Each experiment was performed at least triplicate and results are presented as mean ± s.d., Scale bar: 50 μm. Student’s *t*-test was used to analyze the data (***P* < 0.01, ****P* < 0.001).

**Figure S5.** **Generation of NCAPD3^+/-^ mice and Schematic representation of the AOM/DSS procedure.**


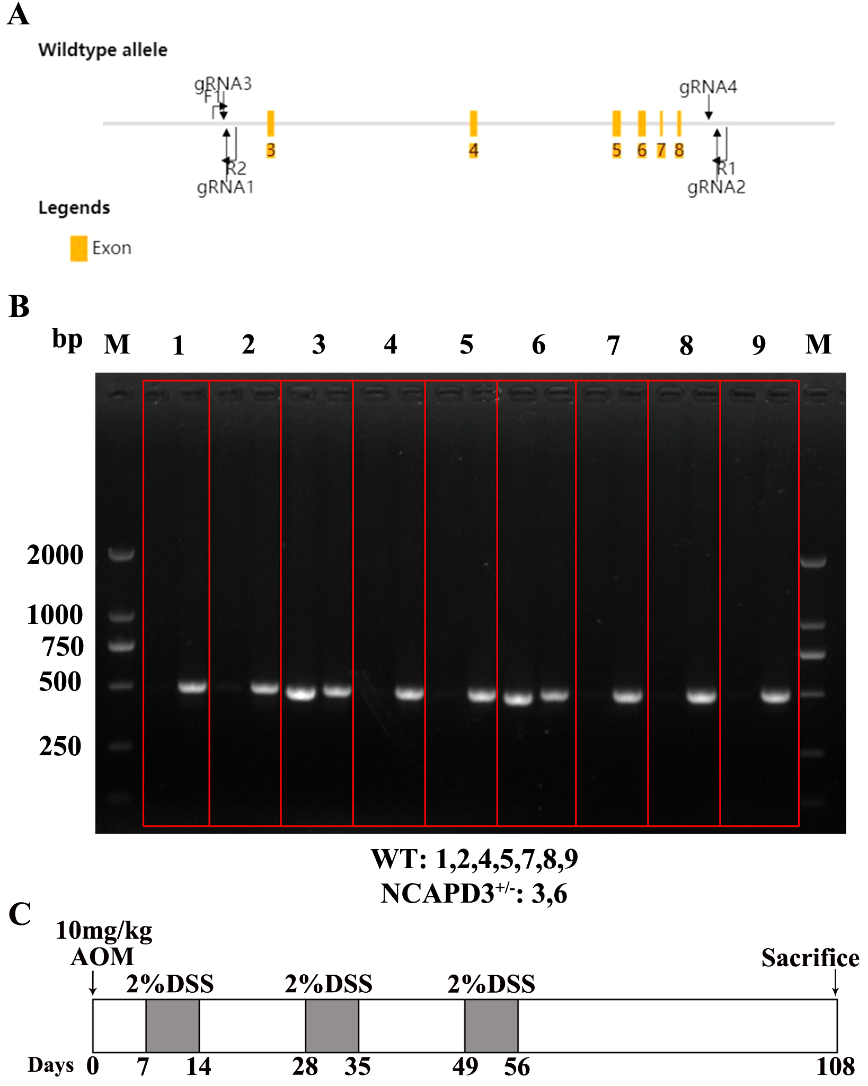


(A) CRISPR targeting strategy: Target sites of sgRNAs are represented by black arrows. (B) The genotyping of NCAPD3 knockout mice was determined by PCR analysis. The PCR product for WT mice is 498 bp. The PCR product for NCAPD3^+/-^ mice is 481 bp and 498 bp. (C) To develop colitis-associated cancer, WT (n = 5) and NCAPD3^+/-^ (n = 5) mice were injected intraperitoneally with AOM (10 mg/kg) on day 0. Then, three cycles of feeding water with 2% DSS treatment were administered. Mice were euthanized on day 108 and intestines were removed and flushed with cold PBS.

**Figure S6. Schematic diagram of NCAPD3 functional and mechanism in CRC.**


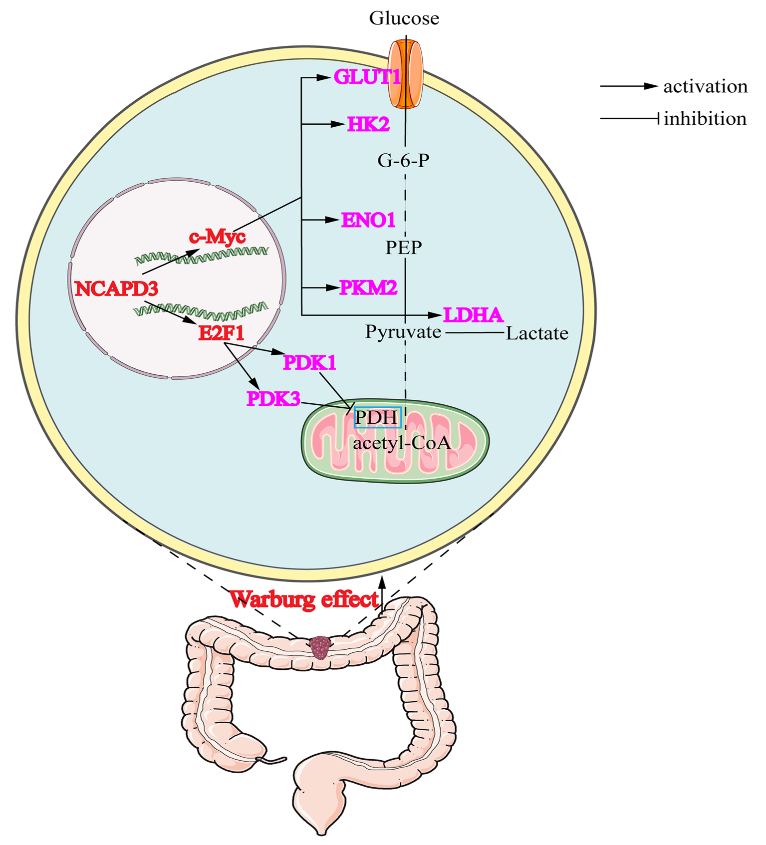


In CRC cells, NCAPD3 interacted with c-Myc which subsequently recruited it to the promoter of downstream glycolytic target genes GLUT1, HK2, PKM2, ENO1 and LDHA. Moreover, NCAPD3 increased the level of E2F1 and recruited it to downstream target genes including PDK1, PDK3. Thus, NCAPD3 promoted the Warburg effect by coordinating glycolysis and TCA cycle to facilitated colorectal cancer development.
