# Supplementary material for: NCAPD3 enhances Warburg effect through c-myc and E2F1 and promotes the occurrence and progression of colorectal cancer
Source: J Exp Clin Cancer Res. 2022 Jun 11;41:198. doi: 10.1186/s13046-022-02412-3 (PMC9188166; doi:10.1186/s13046-022-02412-3)
Supplement: Supplementary file 2 — Additional file 2: Table S1. The sequence of siRNA oligonucleotides targeting NCAPD3, C-myc, E2F1 and negative control. Table S2. Detailed information of antibodies and reagents. Table S3. The primer sequences used for qRT-PCR assay. Table S4. Primer sequences used for ChIP assay. [file 13046_2022_2412_MOESM2_ESM.docx]

**Supplementary tables**

**Table S1. The sequence of siRNA oligonucleotides targeting NCAPD3, C-myc, E2F1 and negative control.**

| **siRNA** | **Sequences** |
| --- | --- |
| NCAPD3 siRNA | AGGAAUUCAAGUUAACAGAGGCUUG |
| negative control siRNA | UUCUCCGAACGUGUCACGUTT |
| c-Myc siRNA | CGAUGUUGUUUCUGUGGAATT |
| E2F1 siRNA | AGAUGGUUAUGGUGAUCAATT |

**Table S2. Detailed information of antibodies and reagents.**

| **Antibodies Catalog No.** | **Manufacturer** |
| --- | --- |
| NCAPD3 sc-101016 | Santa Cruz |
| E2F1 sc-251 | Santa Cruz |
| c-Myc A1309 | ABclonal |
| GLUT1 A11170  HK2 A0994  PKM2 A19102  LDHA A1146  PGK1 A14039  PGAM1 A4015  PDK1 A0834  PDHE1α A1895  β-actin AC006  ENO1 3810T  PDK3 ab154549  p-PDHE1α ab177461  HRP Goat Anti-Rabbit IgG (H+L) AS014  HRP Goat Anti-Mouse IgG (H+L) AS003  HLM006474 HY-16667  10058-F4 HY-12702  Azoxymethane A5486  Dextran sulfate sodium 9011-18-1 | ABclonal  ABclonal  ABclonal  ABclonal  ABclonal  ABclonal  ABclonal  ABclonal  ABclonal  Cell Signaling Technology  Abcam  Abcam  ABclonal  ABclonal  MedChemExpress  MedChemExpress  Sigma  MP Biomedicals |

**Table S3. The primer sequences used for qRT-PCR assay.**

| **Primer** | **Sequences（5’- 3’）** |
| --- | --- |
| β-actin | F-GAGCTACGAGCTGCCTGACG |
|  | R-CCTAGAAGCATTTGCGGTGG |
| NCAPD3 | F-TGGAGCAAGAGTCGAATGGCG |
|  | R-GGGGCGGTTTATCAGGCAGTG |

**Table S4. Primer sequences used for ChIP assay.**

| **Gene** | **Sequences（5’- 3’）** |
| --- | --- |
| GLUT1 | F-TCATGTCCTTAATGTCCTATCATG |
|  | R-CCCGTTTGTGAGCCAAGCCTTGCT |
| HK2 | F-AGGAAGAGCAAAGACCCTTGGGTG |
|  | R-GCCACTCACCCTCACAGCCAGTC |
| ENO1 | F-GTTCTGGCCACAGCAGAGTATCTT |
|  | R-GTAAGGACTCTAAGAAATCAGGCAC |
| PKM2 | F-TACAGGCGTGAGCCACTGCACC |
|  | R-AAAAGACATGCCCCCCTAGGGAG |
| LDHA | F-GATCAGCCTGACCAACATGGTGAAA |
|  | R-TGAAACGGAGTCGCTCTGTCGCC |
| PDK1 | F-TACCCGTTACACTTTCTAAAACCAACA |
|  | R-CCAATCTGCGTTTTCCCTGAA |
| PDK3 | F-GGTGTTATTGACCCTTGAC |
|  | R-CTGACTCTGTTTCGTGGC |
